# Prevalence, Etiology, and Risk Factors Associated with Occurrence of Canine Cutaneous Myiasis in Kitui County, Kenya

**DOI:** 10.1155/2022/5699060

**Published:** 2022-06-24

**Authors:** Kamuti N. Mutinda, Mbuthia P. Gichohi, Waruiru R. Maina, Githigia S. Maina, Keya E. Agosa

**Affiliations:** ^1^Department of Veterinary Pathology, Microbiology and Parasitology, University of Nairobi, P.O. BOX 29053-00625, Kangemi, Nairobi, Kenya; ^2^Department of Veterinary Pathology, Microbiology and Parasitology, Faculty of Veterinary Medicine, University of Nairobi, P.O. Box 29053-00625, Kangemi, Nairobi, Kenya

## Abstract

Myiasis is the infestation of living tissues of animals with dipterous larvae. In Africa, *Cordylobia* species (*C. anthropophaga, C. rodhaini,* and *C. ruandae)* and *Dermatobia hominis* are reported as the principal cause of nonmigratory cutaneous myiasis of domestic animals. None of these have been reported in dogs in Kenya. A cross-sectional study was conducted in eight subcounties of Kitui County, Kenya, from March to August 2021 to estimate the prevalence, risk factors, and etiological agents associated with canine cutaneous myiasis (CCM). A questionnaire was administered to dog owners to collect information on CCM risk factors. A total of 400 dogs were physically examined and larvae collected from myiasis skin lesions and preserved in 70% ethanol, taken to the laboratory, processed and identified using parasitological morphological features. Live larvae were incubated and emerging adults were captured and identified. The overall prevalence of CCM was 45% (180/400) (95% confidence interval: 40.0–50.0%). A total of 434 larvae were collected from 180 dogs infested with cutaneous myiasis. All larvae (100%) were identified as *C. anthropophaga* and hatched adults were “tumbu” flies. There were no significant differences in the prevalence of CCM at 95% confidence interval among different age and sex groups (*p* > 0.05), although puppies (<6 months) appeared more affected. The highest prevalence of myiasis was in Kitui Central at 65% (95% confidence interval: 51.6–76.9%), Mwingi North at 52.5% (95% confidence interval: 36.1–68.4%), Kitui South at 48.5% (95% confidence interval: 31.5–63.9%), Kitui Rural at 40% (95% confidence interval: 27.6–53.5%), Mwingi Central at 40% (95% confidence interval: 24.9–56.7%), Mwingi West at 40% (95% confidence interval: 24.9–56.7%), Kitui West at 38.3% (95% confidence interval: 26.1–51.8%), and Kitui East subcounty at 36.7% (95% confidence interval: 24.6–50.1%). Lack of housing, housing structures, and dog living area environmental hygiene were the main risk factors associated with the occurrence of CCM (*p* < 0.05). The CCM occurrence was significantly different among breeds (*p* < 0.05). *Cordylobia anthropophaga* larvae were the etiological agent of CCM in Kitui County. There is a need for improved dog housing and hygiene measures to prevent the occurrence of CCM, and affected dogs should be treated to prevent the spread of CCM among the dogs.

## 1. Introduction

Myiasis refers to the infestation of the living tissues of humans and other animals with dipterous larvae for a particular duration where they feed on the host's liquid body substances, ingested food, and dead or living tissue [1]. After infestation, larvae complete part of their life cycle in or on the vertebrate body [2].

Cutaneous myiasis can be classified into three categories based on the cutaneous system clinical manifestation of the disease as furuncular, creeping, and wound myiasis [3, 4]. In tropical regions such as Africa and subtropical regions such as South America, nonmigratory cutaneous myiasis of livestock and humans are usually caused by *Cordylobia* spp. (*C. anthropophaga, C. rodhaini,* and *C. ruandae)* and *Dermatobia hominis,* respectively [5–7]. McGraw and Turiansky [8] reported that *C. anthropophaga* is the principal etiological agent of CCM because humans, dogs, and rodents serve as reservoir hosts for the larvae.

Myiasis is more often found in domestic animals in the tropics especially in developing countries where it is associated with poor animal welfare [9]. It is mainly predisposed by a low level of animal hygiene, cage, and environmental hygiene [9]. The condition is fairly common but underestimated in many rural areas [3] despite its great medical and veterinary importance [10]. Myiasis-causing flies are attracted by skin injuries, urine, and feces in the animals' environment. Female flies deposit eggs in skin wounds or on sleeping areas especially on bedding, sand, or straws [11]. The fly eggs hatch and the resulting larvae penetrate the animal skin after coming into direct contact from the environment or bedding where it feeds on dead or living tissues and body fluids in the body of the host animal [12]. The growth rate and development of the larvae from L_1_ to L_3_ stage (first instar to third instar) and the pupal stage is determined by the Diptera fly species, environmental temperature, and to a lesser extent by humidity [12]. Factors that determine the occurrence of clinical myiasis in humans and animals include high ambient temperature, humidity, rainfall intensity, and host susceptibility [13].

Kitui County has semiarid and arid climatic zones with temperatures ranging between 14°C and 32°C [14]. This temperature range is ideal for breeding and spread of dipterous larvae associated with CCM [15]. In Kenya, Obanda et al. [10] reported a case of traumatic myiasis in wild game eland due to *Chrysomia bezziana* and *Lucilia* spp. Mugachia [16] in a newspaper reported observing lesions of CCM in Kitui County. However, Mugachia [16] did not establish the specific etiological agent, risk factors, and prevalence of CCM. Further, there is no published data on the prevalence and risk factors of this condition in livestock and humans in Kenya. The aim of this study was therefore to estimate the prevalence and risk factors and characterize and identify the etiological agent associated with CCM in Kitui County.

## 2. Materials and Methods

### 2.1. Study Area

**Figure 1:**
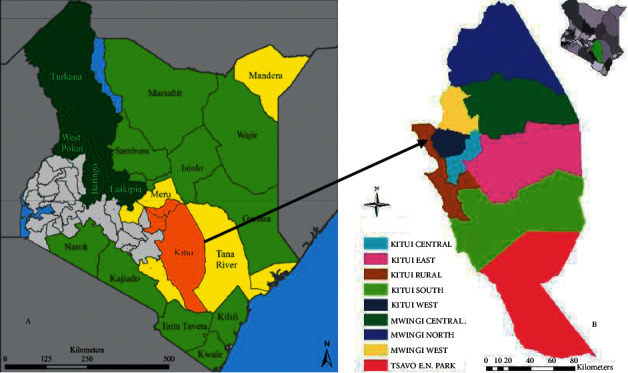
A map of Kenya (a) showing Kitui County (in brown, the study area) and eight subcounties of Kitui County (b) by Klisch and Atzberger [17].

A cross-sectional study was carried out in the eight subcounties of Kitui County (Figure 1). The county is located approximately 160 km from Nairobi between latitudes 0°10 and 3°0 south and longitudes 37°50 and 39°0, with an altitude ranging from 400 to 1800 m above sea level [14]. Selection of the study area was based on the assumption that CCM is endemic in the study area [16]. The county experiences bimodal rain patterns: “short” October to December rains which are more reliable and are the county's principal productive season and “long” March to May rains which are usually unreliable [18].

### 2.2. Target Population and Study Design

Ethical clearance for the use of animals in this study was sought and approved by the Biosafety, Animal Use and Ethics Committee of the Faculty of Veterinary Medicine, University of Nairobi (REF: FVMBAUEC/2021/303). The target animal population were dogs of different breeds, age, and sex from various households in the county to ensure a systematic random selection of sampled animals. During sampling, the selection of the specific wards was based on a list of ongoing antirabies vaccination schedules with concurrent treatment of dogs having cutaneous myiasis during vaccination in the study area. The last ward in each subcounty in the vaccination schedule was selected for sampling. Systematic random selection of the villages and specific households was based on a list kept at the local administrative chief's office. The households were selected at an interval of 5 households.

### 2.3. Sample Size

A 50% expected prevalence was used due to a lack of previous studies in Kitui County on CCM. A 5% precision and a 95% confidence interval were used to determine the sample size using the formulae described by Thrusfield [19]. Four hundred dogs from 248 households were examined and sampled.

### 2.4. Questionnaire Administration

Semistructured questionnaires on biodata of the dog owners and dogs, dog diseases, and risk factors for CCM such as housing, housing hygiene, and frequency of cleaning dog kennels were prepared. The questionnaires were administered through face-to-face interviews with dog owners in the study area. Eighty interviews were conducted from 248 households and responses were recorded in hard copy questionnaires.

### 2.5. Examination of Dogs for Canine Cutaneous Myiasis in Kitui County

#### 2.5.1. Selection of Sampled Dogs

Physical examination of the dogs' skin was conducted and infested dogs were sampled at selected households whose owners were available during the household visits and willing to have their dogs examined and sampled in the county. A maximum of three dogs of different ages or breeds and either sex were examined and sampled per household to ensure random selection of the dogs because some households owned more than three dogs of different breeds, ages, and sex groups. Dogs aged below 6 months were considered as puppies, 6 months to 12 months as young adults, and above 12 months as adults [19]. The approximate age of the dogs in households where the age of the dogs was not indicated on the vaccination card records was determined by examining their teeth as described by the Humane Society of the United States [20].

#### 2.5.2. Restraint, Physical Examination, and Sampling of the Dogs

Four hundred dogs were examined and those with CCM were sampled from all eight subcounties of Kitui County. Based on the dog owners' willingness to participate in the study questionnaires and verbal consent to have their dogs examined for myiasis skin lesions and to a greater extent the resources available for this study, sixty dogs were physically examined in each of the subcounties of Kitui Central, Kitui West, Kitui Rural, and Kitui East whereas only forty dogs were examined from each of the four subcounties of Kitui South, Mwingi Central, Mwingi North, and Mwingi West (Table 1).

During sampling, particulars of the dogs such as the sex, age, and breed were recorded.

Puppies and young adults were restrained using small-sized dog muzzles by the dog owners for physical examination of their skin for the presence of myiasis lesions. Young adults and adult dogs were restrained using a leash and medium- and large-sized dog muzzles depending on the size of the dogs for physical examination of their skin for the presence of CCM lesions. At least two larvae were harvested by the application of gentle digital pressure at the base of the CCM lesions to express the dipterous larvae. The harvested larvae were immediately preserved in 70% ethyl alcohol and transported in cool boxes to the Department of Veterinary Pathology, Microbiology, and Parasitology, University of Nairobi, for characterization and identification using standard laboratory techniques [21].

### 2.6. Laboratory Processing of Larvae for Parasitological Features Identification

In the laboratory, the larvae were washed in distilled water, cleared in 10% sodium hydroxide solution for one hour, washed in distilled water, transferred to 10% acetic acid for 30 minutes, and finally washed in distilled water. They were then dehydrated in ascending concentrations of ethanol (30%, 50%, 70%, and 90%) for thirty minutes in each solution. Thereafter, they were then soaked in absolute alcohol (100%) for one hour and cleaned in xylene for one hour. All the internal organs of the maggots were then removed and the posterior spiracles were cut transversely, the anterior end was cut transversely, and the thoracic and abdominal body segments were cut longitudinally. The specimens were then mounted onto glass slides using DPX mountant and dried in an oven at a temperature of 38°C for 48 hours [22]. After drying, the mounted specimens were then examined under an Olympus digital microscope for characteristic parasitological morphological features and identification of the fly larvae.

### 2.7. Culturing of *Cordylobia anthropophaga* Larvae to Adult Flies

An aluminum foil lining was applied at the base of a plastic dish, and dry sand was applied to the plastic dish. A piece (0.25 kg) of fresh goat liver was then put into the plastic dish container for the dipteran maggots to feed on during their development and the incubation period before they pupate. Ten live dipteran maggots were freshly collected from the skins of dogs with CCM. The *C. anthropophaga* larvae were then put into the plastic dish container. The plastic dish container was covered using cotton gauze and placed in the open environment in Umuu village, Changwithya East Ward, Kitui Central Subcounty, at ambient temperature and exposure to direct sunlight. The development of the maggots into various stages was monitored for 4 weeks [21].

### 2.8. Statistical Data Analysis

Raw data were recorded in the Microsoft Excel 2016 spreadsheet and descriptive statistics analysis was carried out using Microsoft Excel 2016. Categorical variables of the dogs, namely, breed, sex, age, and housing, were expressed as a percentage.

The prevalence of CCM was calculated using the following formula [23]:

(1) $$\begin{array}{c} \frac{\text{total number of dogs infested with CCM}}{\text{total number of dogs examined}}. \end{array}$$

Fisher's exact test was used for the analysis of any relationship linking the categorical variables of the sampled dogs, namely, age, sex, breed, risk factors such as housing and environmental hygiene, and the prevalence of CCM in the county. These variables were considered significant at *p* < 0.05.

## 3. Results

### 3.1. Prevalence of Canine Cutaneous Myiasis

The overall prevalence of CCM in Kitui County was 45% (95% confidence interval: 40.0–50.0%), with the highest prevalence recorded in Kitui Central at 65% (95% confidence interval: 51.6–76.9%) and the lowest reported in Kitui East at 36.7% (95% confidence interval: 24.6–50.1%) (Table 2).

### 3.2. Gender, Breed, and Age Prevalence of Canine Cutaneous Myiasis

The prevalence of CCM in males and females was 42.7% (95% confidence interval: 36.3–49.2%) and 48.5% (95% confidence interval: 40.5–56.4%), respectively, in Kitui County (Table 3). The prevalence of CCM in puppies (<6 months), young adults (6–12 months), and adults (>12 months) was 51.1% (95% confidence interval: 36.1–65.9%), 34.6% (95% confidence interval: 22.0–49.1%), and 45.9% (95% confidence interval: 40.1–51.7%), respectively (Table 3). However, differences in sex and age groups were not statistically significant (*p* > 0.05). The prevalence of CCM in local (mongrels) and pure-breed dogs was 47.5% (95% confidence interval: 41.5–51.9%) and 8% (95% confidence interval: 40.98–2.6%), respectively (Table 3). This difference was statistically significant (*p* > 0.05).

### 3.3. Relationship between Housing Structures, Their Hygiene, and Occurrence of Canine Cutaneous Myiasis in Kitui County

The prevalence of canine cutaneous myiasis in the housed dogs and lairs was 2.0% (95% confidence interval: 0.24–7.1%) and 59.3% (95% confidence interval: 53.5–64.9%), respectively. The difference between the overall prevalence of canine cutaneous myiasis in the housed dogs and lairs was statistically significant (*p* > 0.05; Table 4).

The prevalence of canine cutaneous myiasis in dogs housed in clean kennels (cleaned-daily) was 2.67% (95% confidence interval: 0.3–9.3%), whereas the prevalence in dogs kept in rarely cleaned lairs environment was 54.77% (95% confidence interval: 49.2–60.3%). This difference was statistically significant (*p* > 0.05; Table 4).

### 3.4. Demographic Characteristics of the Human Respondents in Sampled Households in Kitui County

Of the eighty (80) questionnaire respondents, 61.3% were males whereas 38.8% were females. Eighty (80; 100%) of the respondents were dog owners and were aware about canine cutaneous myiasis in Kitui County (Table 5). Knowledge on the etiological agents associated with canine cutaneous myiasis in Kitui County was very low, 16.3% of the respondents were aware about the dipteran flies associated with canine cutaneous myiasis, whereas 83.8% had no idea about the causative agent of canine cutaneous myiasis in Kitui County (Table 5).

### 3.5. Characterization and Identification of Etiological Agent Associated with Canine Cutaneous Myiasis

#### 3.5.1. Macroscopic Appearance of the Dipterous Larvae

**Figure 2:**
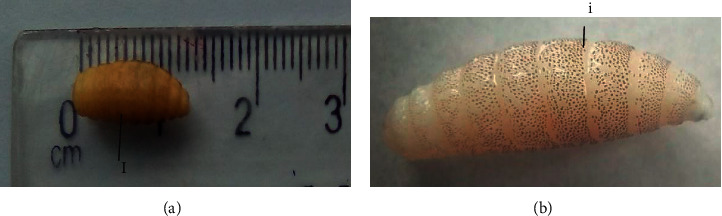
(a) Length of *Cordylobia anthropophaga* larvae in millimeters (12 mm); (b) *Cordylobia anthropophaga* larvae with a barrel-shaped body with dorsal minute brown cuticular spines on the thoracic and abdominal body segments.

The dipteral larvae were white to dark brown ranging between 8 and 12 mm in length, barrel-shaped, pointed anteriorly, and tapering to a larger posterior end (Figure 2(a)). On the anterior end, the larvae had a pair of black oral hooks. The thoracic and abdominal body segments had brown minute cuticular spines (Figure 2(b)). The posterior end had two posterior spiracles not widely separated.

#### 3.5.2. Microscopic Appearance of the Mounted Sections of the Dipterous Larvae

**Figure 3:**
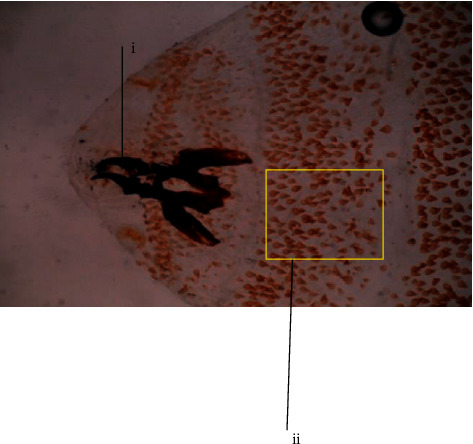
The anterior end of *Cordylobia anthropophaga* larvae showing pair of oral hooks (i) and short conical brown cuticular spines (ii) on the thoracic body segments (×40 magnification).

The anterior end of *C. anthropophaga* larvae had a cephalopharyngeal skeleton with a pair of oral hooks (i) and short brown conical cuticular spines (ii) on the thoracic body segments of the larvae (Figure 3).

**Figure 4:**
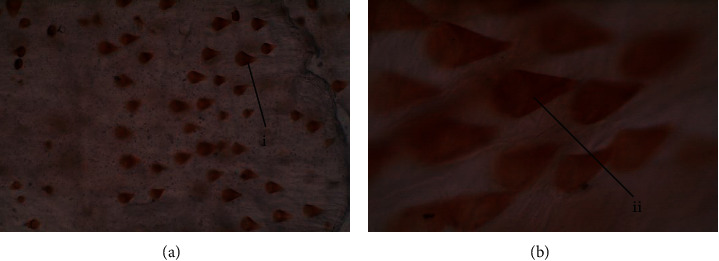
Thoracic and abdominal body segments' brown conical cuticular spines. (a) ×40 magnification; (b) ×100 magnification.

The thoracic and abdominal body segments of the *C. anthropophaga* larvae had posteriorly pointed brown conical cuticular spines (Figure 4).

**Figure 5:**
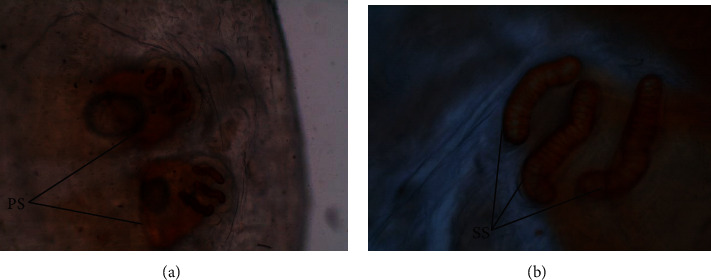
Posterior end of *Cordylobia anthropophaga* larvae with two posterior spiracles (PS) each with three sinuous spiracular slits (SS). (a) ×40 magnification; (b) ×100 magnification.

The *C. anthropophaga* larvae on the posterior end had two posterior spiracles each with three distinct spiracular slits (Figure 5).

### 3.6. Culture *Cordylobia anthropophaga* Larvae

**Figure 6:**
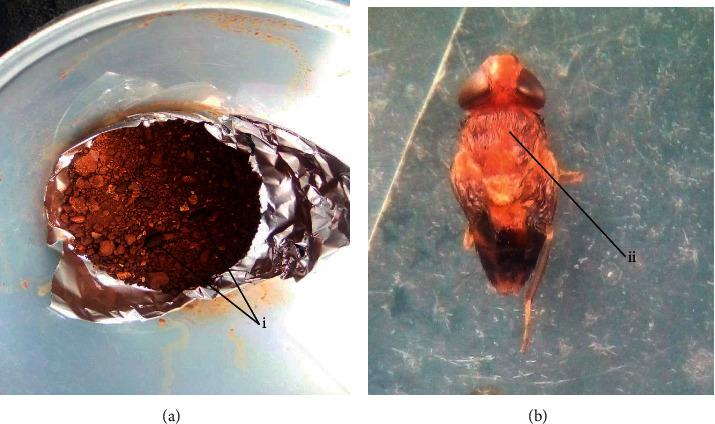
Pupae (a) (i) and adult tumbu fly (b) (ii) of *Cordylobia anthropophaga* after culturing of larvae at ambient temperature and exposure to direct sunlight in the field.

On culturing, the larvae pupated and hatched into adult “tumbu” flies. These were yellow-brown in color with diffuse bluish-grey patches on the thorax and dark grey color on the posterior part of the abdomen (Figure 6).

## 4. Discussion

The prevalence of CCM in Kitui County, Kenya, was 45% (95% confidence interval: 40.0–50.0%). Kitui County is a semiarid county characterized by high rainfall variability, prolonged droughts, and high temperatures, which could favor the lifecycle of the Diptera flies and the eventual spread of CCM. There is no published data in Kenya to compare with, but our findings were similar to the study by Abebe [24], who reported an overall prevalence of 42.2% in dogs in Ethiopia. They, however, differ from other studies that reported a prevalence of CCM of 7.4% [15] and 75.8% [25] in Ghana and Nigeria, respectively. These differences in prevalence could be attributed to variations in geographical zones and prevailing climatic conditions. Canine cutaneous myiasis prevalence of 45% (95% confidence interval: 40.0–50.0%) in this County might be an underestimation of the condition because only 11.75% of the physically examined dogs in the study area were below 6 months of age. Yet young dogs have been reported before to be more susceptible to CCM [26].

The prevalence of canine cutaneous myiasis in male dogs was slightly lower at 42.7% (95% confidence interval: 36.3–49.2%) than in females at 48.5% (95% confidence interval: 40.5–56.4%) though the difference was not significant (*p* > 0.05). This differed from Abebe [24], who reported a sex predisposition to CCM in Ethiopia.

This condition was more prevalent in the local (mongrels) than in pure-breed dogs at 47.5% (95% confidence interval: 41.5–51.9%), suggesting breed predisposition (*p* < 0.05). These findings compared with previous studies by Johnson et al. [15] who reported an 82.8% prevalence among mongrels in Greater Accra in Ghana. However, they differed from Abebe [24], who reported that there was no breed predisposition to CCM in Ethiopia. Low prevalence of canine cutaneous myiasis in pure breeds may be attributed to the fact that they are given special care such as proper housing and routine hair coat grooming to control ectoparasites using pour-on formulations and dog shampoos [27].

Young puppies (<6 months) were the most infested age group at 51.6% (95% confidence interval: 36.1–65.9%), which is in agreement with previous studies [15, 24, 25] that reported a prevalence of 68.95%, 93.1%, and 71.56%, respectively. Abebe [24] reported that low prevalence of CCM in adult dogs is attributed to low exposure to the ground where the eggs of “tumbu fly” are found unlike older dogs. Routine contact with the ground and associated higher heat dissipation may attract the fly larvae, hence infestations.

Lack and type of housing and poor hygiene measures were the major risk factors for the exposure and occurrence of CCM in dogs in the study area (*p* < 0.05). The prevalence of canine cutaneous myiasis was higher in unhoused dogs at 59.3% (95% confidence interval: 53.5–64.9%) where the environment was rarely cleaned and was dirty during sampling. This was in agreement with the previous study by Johnson et al. [15] who stated that unhoused dogs were more likely to easily move about in the community in search for food and could be exposed to the dipterous flies leading to the development of myiasis. Earlier, Yanuartono et al. [9] reported that dogs are predisposed to myiasis by a low level of animal hygiene and cage and environmental hygiene. Further, Anderson and Huitson [28] reported that neglected pets have an accumulation of urine and feces which is one of the primary risk factors for the development of CCM.

All respondents were dog owners who were aware of the occurrence of CCM in the County. However, very few knew the etiological agents associated with the disease at 16.25%, while others were unaware of the dipteran flies associated with CCM at 83.75%. These findings suggest that lack of knowledge on the etiological agents and poor control measures play a role in the high prevalence of CCM in the study area.

Low prevalence of CCM was reported in dogs where the owners regularly controlled ectoparasites such as fleas and ticks through routine application of dog shampoos and pour-on formulations compared with dogs where the owners never applied any control measures. This was in agreement with previous studies by Johnson et al. [15] who reported that dogs whose owners controlled ectoparasites on their dogs on a regular basis were least infested compared with those receiving bimonthly and quarterly ectoparasite control.

These findings are in agreement with earlier work by Ogo et al. [29] and Abebe [24], who reported 100% *C. anthropophaga* larvae infestation associated with CCM in Nigeria and Ethiopia, respectively. They, however, differ from previous work by Johnson et al. [15], who reported *C. rodhaini* and *Dermatobia hominis* as the principal etiological agents of CCM.

The main limitations of this study were the unwillingness of dog owners to participate in the study questionnaire interviews and limited funding. The dog owners should be enlightened to participate in future research studies in the study area.

Dogs play a major role as reservoirs for *C. anthropophaga* larvae, which are responsible for CCM. Lack of awareness of the etiological agents and control measures for this disease may contribute to the high prevalence of CCM myiasis cases in the study area. Canine cutaneous myiasis is a zoonotic disease and there is a need for further studies on the knowledge, attitude, and practices of the residents in the study area to human cutaneous myiasis.

## 5. Conclusions and Recommendations

The results of the present study revealed that CCM is widespread in the study area and that dogs are housed in different housing structures with different hygienic measures with less emphasis on the predisposing factors. This may be attributed to a lack of knowledge about the etiological agents and control measures for canine cutaneous infestation. *Cordylobia anthropophaga*, the “tumbu” fly or “mango” fly larvae, is the etiological agent for CCM.

It is recommended that there be creation of awareness by veterinarians and public health officers on the etiological agents, risk factors, and control measures of CCM in Kitui County to ensure wellbeing of the host animals. Further studies on seasonal prevalence, different ecological zones and etiological agents in other counties, public health importance, and control strategies of CCM in Kenya are also recommended.

## Figures and Tables

**Table 1:** Number of dogs examined for myiasis lesions in Kitui County

Subcounty	Number of dogs examined
Kitui Central	60
Mwingi North	40
Kitui South	40
Kitui Rural	60
Mwingi Central	40
Mwingi West	40
Kitui West	60
Kitui East	60
Total	400

**Table 2:** Prevalence of canine cutaneous myiasis in eight subcounties of Kitui County.

Subcounty	Number of dogs examined	Number of positive cases (% prevalence)
Kitui Central	60	39 (65)
Mwingi North	40	21 (52.5)
Kitui South	40	19 (48.5)
Kitui Rural	60	24 (40)
Mwingi Central	40	16 (40)
Mwingi West	40	16 (40)
Kitui West	60	23 (38.3)
Kitui East	60	22 (36.7)
Total	400	180 (45)

**Table 3:** Canine cutaneous myiasis in different sex, age, and breed groups of dogs.

Variable	Categorical variable	Number of dogs examined	Positive cases (%)	*p* value
Sex	Male	239	102 (42.7)	0.2616
	Female	161	78 (48.5)	
Age	<6 months	47	24 (51.1%)	0.2182
	6–12 months	52	18 (34.6%)	
	>12 months	301	138 (45.9%)	
Breed	Local/mongrels	375	178 (47.5%)	0.0001
	Pure breeds	25	2 (8%)	

**Table 4:** Prevalence of canine cutaneous myiasis in different housing structures and hygienic measures.

Categorical variable	Q. variable	No. examined	Positive (%)	*p* value
Are the dogs housed?	No	300	178 (59.33)	0.00001
	Yes	100	2 (2)	

Frequency of cleaning the dog house	Daily (clean)	75	2 (2.67)	0.00001
	Rarely (dirty)	325	178 (54.77)	

**Table 5:** Demographic characteristics of the human respondents in sampled households in Kitui County.

Categorical variable	Qualitative variable	Number	Percentage
Sex	Male	49	61.25
	Female	31	38.75
Dog ownership	Yes	80	100
	No	0	0.00
CCM awareness	Yes	80	100
	No	0	0.00
Awareness on the	Flies	13	16.25
Causative agent of CCM	No idea	67	83.75

## Data Availability

Any additional data are available upon request via the corresponding author Dr. Nichodemus Mutinda Kamuti through nkamuti@uonbi.ac.ke.
